# Entomological surveillance of *Trypanosoma cruzi* vectors in Rio Grande do Norte, Brazil

**DOI:** 10.1111/mve.70093

**Published:** 2026-06-26

**Authors:** Letícia Mikardya Lima Sales, Christiane Carlos Araújo de Negreiros, Lucas Abrantes Batista, George Harisson Felinto Sampaio, Nathan Ravi Medeiros Honorato, Carlos Ramon do Nascimento Brito, Rand Randall Martins, Lúcia Maria Abrantes Aguiar, Lúcia Maria da Cunha Galvão, Antonia Claudia Jácome da Câmara, Andressa Noronha Barbosa‐Silva Carvalho

**Affiliations:** ^1^ Graduate Program in Pharmaceutical Sciences, Center for Health Sciences Federal University of Rio Grande do Norte Natal Brazil; ^2^ Graduate Program in Health Sciences Federal University of Rio Grande do Norte Natal Brazil; ^3^ Graduate Program in Parasitology Federal University of Minas Gerais Belo Horizonte Brazil; ^4^ Graduate Program in Parasitic Biology Federal University of Rio Grande do Norte Natal Brazil; ^5^ Government of the State of Rio Grande do Norte State Department of Public Health of Rio Grande do Norte Natal Brazil

**Keywords:** entomological indicators, natural infection, triatomines, vector control

## Abstract

In Rio Grande do Norte, Brazil, updated studies on synanthropic triatomines are needed to elucidate population dynamics and identify high‐risk areas for parasite transmission. This study evaluated active searching, vector control, and entomological indicator activities, as well as the distribution of triatomine species captured in domestic environments across different mesoregions of the state from 2005 to 2022. Secondary data on triatomine captures in domiciliary units (DUs) from municipalities in the Agreste, Central, and West mesoregions were analysed. These data were provided by the Rio Grande do Norte State Secretariat for Public Health. Entomological surveillance activities were implemented discontinuously throughout the period, ranging from 2 to 12 years. No entomological surveillance activities were performed in the East mesoregion, nor in 46.5% (20/43) of municipalities in the Agreste, 18.9% (7/37) in the Central, and 11.3% (7/62) in the West mesoregions. Triatomine presence was recorded in most municipalities where entomological surveillance activities were conducted, and the number of surveyed and positive DUs was higher in the Central and West mesoregions. The overall infestation index was highest in the West and Central mesoregions municipalities. Additionally, the natural infection index by *Trypanosoma cruzi* (Trypanosomatida: Trypanosomatidae) was elevated in all three mesoregions. *Triatoma brasiliensis* and *Triatoma pseudomaculata* were the most frequently captured species. The West mesoregion exhibited the highest number of specimens captured and the greatest diversity of triatomines. Discontinuities in entomological surveillance and vector control, combined with insufficient systematic monitoring and limited integration between the community and health services, likely contributed to persistent infestation and colonization of anthropic environments by triatomines. Furthermore, the constant presence of native triatomine species infected with *T. cruzi* in dwellings underscores the ongoing risk of parasite transmission to humans and domestic animals.

## Graphical abstract

- Discontinuous entomological surveillance and persistent infestation by triatomines highlight the risk of *Trypanosoma cruzi* transmission.
- The diversity of triatomine species in the peridomicile shows the potential for colonization into the home.
- The wide distribution of *Triatoma brasiliensis* and *Triatoma pseudomaculata* accompanies high rates of infection.

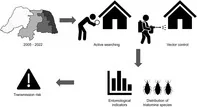

## INTRODUCTION

Chagas disease (CD) is a neglected endemic disease caused by the protozoan *Trypanosoma cruzi* Chagas (Trypanosomatida: Trypanosomatidae). Its infection is endemic in 21 Latin American countries (World Health Organization, 2015) and approximately 10.5 million individuals are estimated to be infected worldwide, mainly in this area (Cousin et al., 2025). In addition, 100 million people live in areas at risk of acquiring infection (Cousin et al., 2025). Approximately 1 million people are infected in Brazil, mainly in rural areas due to the presence of vectors in the domestic environment (Brasil, Ministério da Saúde, 2024).

The vectors of *T. cruzi* are triatomines, haematophagous insects belonging to the subfamily Triatominae (Hemiptera: Reduviidae), currently comprising 159 species, including three fossil species (Barbosa‐Silva et al., 2026; Gil‐Santana et al., 2022; Paiva et al., 2022; Téllez‐Rendón et al., 2023; Zhao et al., 2023). According to Costa et al (Costa et al., 2021), new triatomine species have been frequently described in several countries, especially in Brazil. The most recently described species was *Triatoma chiarii* in the state of Rio Grande do Norte (RN) (Barbosa‐Silva et al., 2026). To date, more than 60 species of triatomines have been recorded in Brazil, of which approximately 20 are of epidemiological importance due to their involvement in the domestic and peridomestic transmission cycle of the parasite, high susceptibility to *T. cruzi* infection, and ability to invade and colonise artificial ecotopes (Costa et al., 2021; Galvão et al., 2014; Gurgel‐Gonçalves et al., 2012; Parente et al., 2017).

Triatomine fauna in the state of Rio Grande do Norte (RN) is quite diverse, mainly consisting of the species *Triatoma brasiliensis* Neiva, *Triatoma pseudomaculata* Corrêa & Spínola, *Panstrongylus lutzi* Neiva & Pinto, *Panstrongylus megistus* Burmeister and *Rhodnius nasutus* Stal (Costa et al., 2003; Lucena, 1959; Silveira & Dias, 2011). *Triatoma brasiliensis* has been considered the main vector of *T. cruzi* in this state since the 1950s due to its presence in wild ecotopes in rocky outcrops, as well as in peridomestic annexes and indoors (Araújo‐Neto et al., 2019; Barbosa‐Silva et al., 2016; Bezerra et al., 2020; Honorato et al., 2021; Lilioso, Reigada, et al., 2020; Lucena, 1959). This species, along with *T. pseudomaculata*, has been highlighted due to the frequency with which they have been found infesting and colonising domiciliary units (DUs) in the state, often presenting high indices of natural infection by the parasite (Barbosa‐Silva et al., 2016, 2019; Honorato et al., 2021; Lilioso et al., 2017; Lilioso, Reigada, et al., 2020; Lima‐Neiva et al., 2021). A recent study documented interactions between *T. brasiliensis* and humans in natural environments frequented for tourism in Rio Grande do Norte, underscoring the urgent need for targeted public awareness campaigns aimed at preventing *T. cruzi* transmission in these accessible ecosystems and reducing opportunities for vector–human contact (Pires‐Silva et al., 2025).

Chemical control of vectors is the main action for reducing the vectorial transmission of the parasite. However, entomological surveillance and chemical control activities, which have a low residual effect (Coutinho et al., 2014), were carried out intermittently in the DUs in RN. Consequently, inconsistency in executing these measures favours the occurrence of domiciliated vectors with a high index of natural infection by *T. cruzi* in anthropic environments (Barbosa‐Silva et al., 2019; Sampaio et al., 2023). In the state of RN, several studies have demonstrated the continuous presence of synanthropic triatomines infesting and colonizing DUs across multiple municipalities, with elevated infection percentage reported for different triatomine species (Araújo‐Neto et al., 2019; Barbosa‐Silva et al., 2016, 2019; Câmara et al., 2010; Honorato et al., 2021; Lilioso, Reigada, et al., 2020; Lima‐Neiva et al., 2021; Sampaio et al., 2023). Additionally, the association of *T. brasiliensis* with infected dogs, considered sentinels for *T. cruzi* infection (Araújo‐Neto et al., 2019; Castillo‐Neyra et al., 2015), as well as other animals has been reported (Honorato et al., 2021; Lilioso, Reigada, et al., 2020).

Accordingly, integrative studies encompassing entomological surveillance, assessment of entomological indicators, and spatial analysis of vector distribution are scarce in RN and are critical to elucidating the current dynamics of triatomine populations and to identifying areas at greatest risk for parasite transmission. Thus, entomological surveillance, active searching, vector control, and entomological indicator activities, in addition to the distribution of triatomine species captured in artificial environments, were evaluated in the different mesoregions of the state of RN from 2005 to 2022. This knowledge is fundamental to epidemiological monitoring, enabling the assessment of control measure effectiveness, the establishment of entomological surveillance priorities, and the development of strategic frameworks in public health.

## METHODS

### Study area

The state of RN is located in the Northeast region of Brazil, has a territorial extension of 52,809.6 km^2^ and is composed of 167 municipalities grouped into four mesoregions: West, Central, Agreste and East (Figure 1a). The predominant climate is semi‐arid and the Biome is Caatinga (Instituto de Desenvolvimento Sustentável e Meio Ambiente do Rio Grande do Norte (IDEMA), 2022).

**FIGURE 1:**
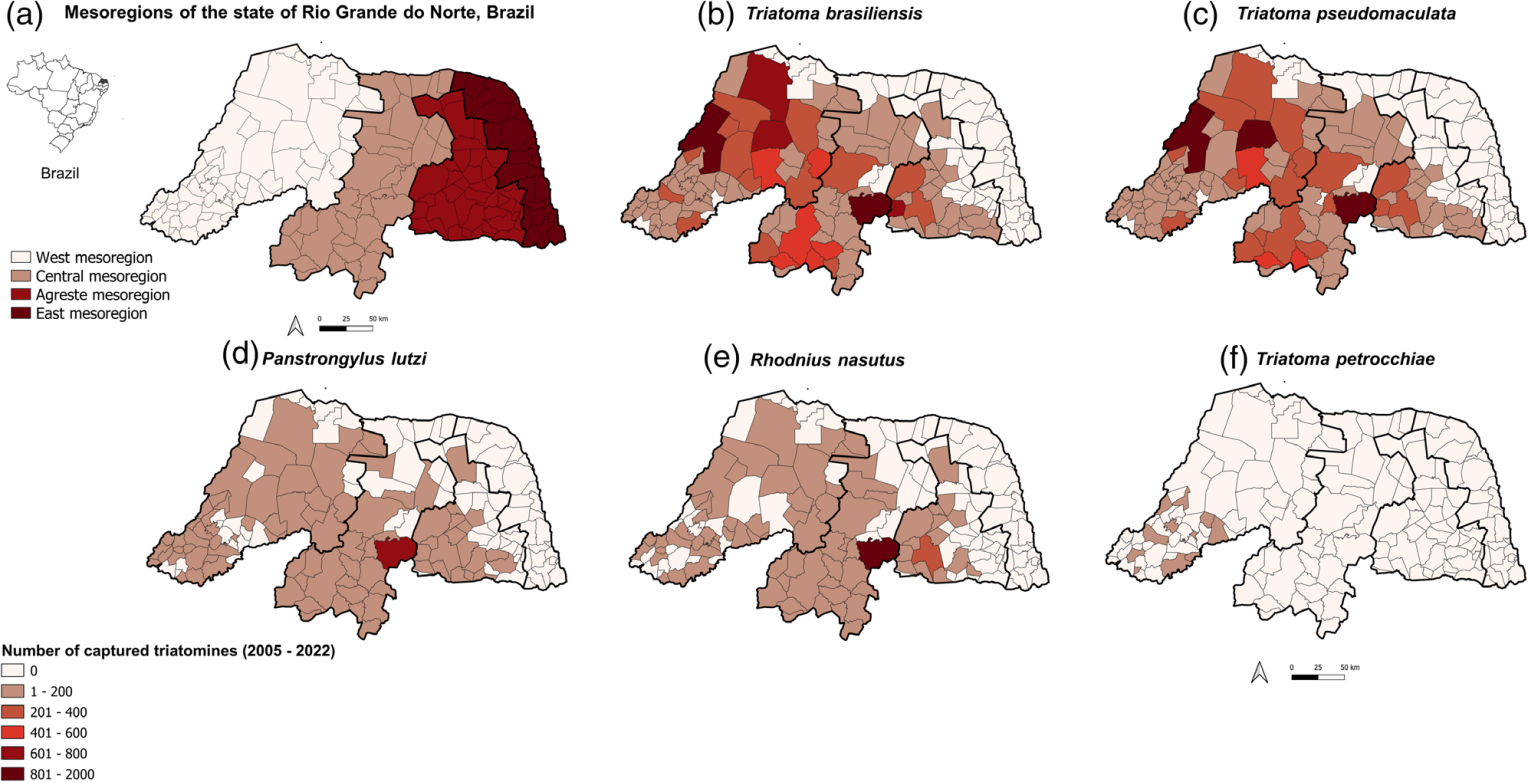
(a) Map of the state of Rio Grande do Norte highlighting the West, Central, Agreste and East mesoregions. (b–f) Distribution of the species of triatomines captured in domiciliary units of different municipalities in the State of Rio Grande do Norte, Brazil from 2005 to 2022.

According to data related to the risk stratification for *T. cruzi* transmission organized by the Ministry of Health in 2012 and reported by Barbosa‐Silva et al. (2019). RN has municipalities considered to be at high risk, medium risk, and low risk of transmission. In this study, municipalities from the West, Central, and Agreste mesoregions of RN were included based on the availability of data regarding entomological surveillance and vector control activities for triatomines. Municipalities in the East mesoregion were excluded because these activities are not routinely conducted, preventing the acquisition of consistent information necessary for indicator analysis and comparison. Data from the years 2016, 2017, and 2018 were not analysed due to limitations in resources, whereas data from 2020 and 2021 were unavailable as a result of the COVID‐19 pandemic and interruptions in entomological surveillance activities.

### Entomological data

Secondary data related to active surveillance and vector control activities for triatomines were analysed, including records of captures in DUs from 2005 to 2022. These data were provided by the State Department of Public Health, which is responsible for managing the information collected by the municipalities which perform epidemiological surveillance activities. Triatomine species were identified using the taxonomic key proposed by Lent and Wygodzinsky (Lent & Wygodzinsky, 1979). Parasitological examination of the specimens was performed through abdominal compression, followed by optical microscopic analysis of the faecal material. These procedures were carried out by trained technicians from the Regional Units of Public Health of the State.

The following entomological indicators were calculated as recommended by the World Health Organization (World Health Organization, 2024): (i) infestation index (number of positive DUs × 100/number of DUs surveyed); (ii) intradomiciliary infestation index (number of positive intradomiciliary DUs × 100/number of intradomiciliary units examined); (iii) peridomiciliary infestation index (number of positive peridomiciliary units × 100/number of peridomiciliary units surveyed); (iv) colonization index (number of DUs with nymphs in the intradomicile×100/number of DUs with triatomines); and (v) natural infection index by *T. cruzi* (number of infected triatomines × 100/number of insects examined). The intradomicile area corresponds to the house interior where the inhabitants sleep, and the peridomicile area corresponds to the area surrounding the house, which is affected by human activities (Bos, 1988).

### Statistical analysis

The statistical analysis included an initial descriptive phase in which the presence of different triatomine species was reported by municipality and mesoregion using absolute and relative frequencies. Data referring to the five entomological indicators were subjected to statistical analyses for regional comparisons and temporal trends. One‐way analysis of variance (ANOVA) was performed to compare each indicator across the three mesoregions of the state of RN (Agreste, Central, and West), followed by Bonferroni post‐hoc tests to identify statistically significant differences between mesoregion pairs (*p* < 0.05). Assumptions of residual normality and homogeneity of variances across groups were assessed in advance to ensure the validity of the parametric tests. All analyses were conducted using Stata software, version 17 (StataCorp, College Station, TX, USA).

In turn, linear mixed‐effects models were fitted to evaluate the temporal trends of each indicator. Each entomological indicator was treated as the dependent variable, while time (in years) was included as a fixed effect. Municipality‐level variability was modelled as a random effect to account for the correlation of repeated measurements within municipalities over the years. Statistical significance was set at *p* < 0.05.

Based on the entomological surveillance database, the geographic distribution of triatomine species in the state of RN was analysed through geospatial mapping. The number of insects captured per municipality was used to generate thematic maps representing the spatial patterns of vector occurrence and species diversity over the study period. Maps were created using QGIS software, version 3.16 (QGIS Geographic Information System, 2020).

## RESULTS

The frequency of entomological surveillance activities in the different mesoregions of RN from 2005 to 2022 can be observed in Table S1. Continuous entomological surveillance was conducted in 14.5% (9/62) of the municipalities in the West mesoregion, 10.9% (4/37) of the municipalities in the Central mesoregion and 4.7% (2/43) of the Agreste mesoregion. However, these activities were not performed in the municipalities in the East mesoregion (data not shown), nor in 46.5% (20/43), 18.9% (7/37) and 11.3% (7/62) of the municipalities in the Agreste, Central and West mesoregions, respectively. The presence of triatomines was documented in most municipalities that conducted entomological surveillance activities: 53.5% (23/43) in the Agreste mesoregion, 81.1% (30/37) in the Central mesoregion, and 96.8% (60/62) in the West mesoregion.

The number of surveyed and positive DUs was higher in the Central and West mesoregions. The number of sprayed DUs in the Agreste mesoregion was lower than the number of positive DUs, accounting for 63.6% (Table S2). Regarding entomological indicators, the overall infestation index was higher in municipalities of the Central (4.3%) and West mesoregions (5.5%) (Figure 2a). The intradomiciliary infestation index was highest in the Central mesoregion (8.2%), followed by the West mesoregion (2.2%) (Figure 2b). In contrast, the peridomiciliary infestation index reached its highest value in the Agreste mesoregion (13.3%), with lower averages observed in the West (8.7%) and Central (8.0%) mesoregions (Figure 2c). Colonization indices were elevated across all three mesoregions, with averages of 25.3% in the Agreste, 29.1% in the West, and 14.2% in the Central mesoregion (Figure 2d). It is important to note that the natural infection index by *T. cruzi* was higher in the Central (4.3%) and Agreste (2.5%) mesoregions compared to the West mesoregion (1.9%) throughout the study period (Figure 2e).

**FIGURE 2:**
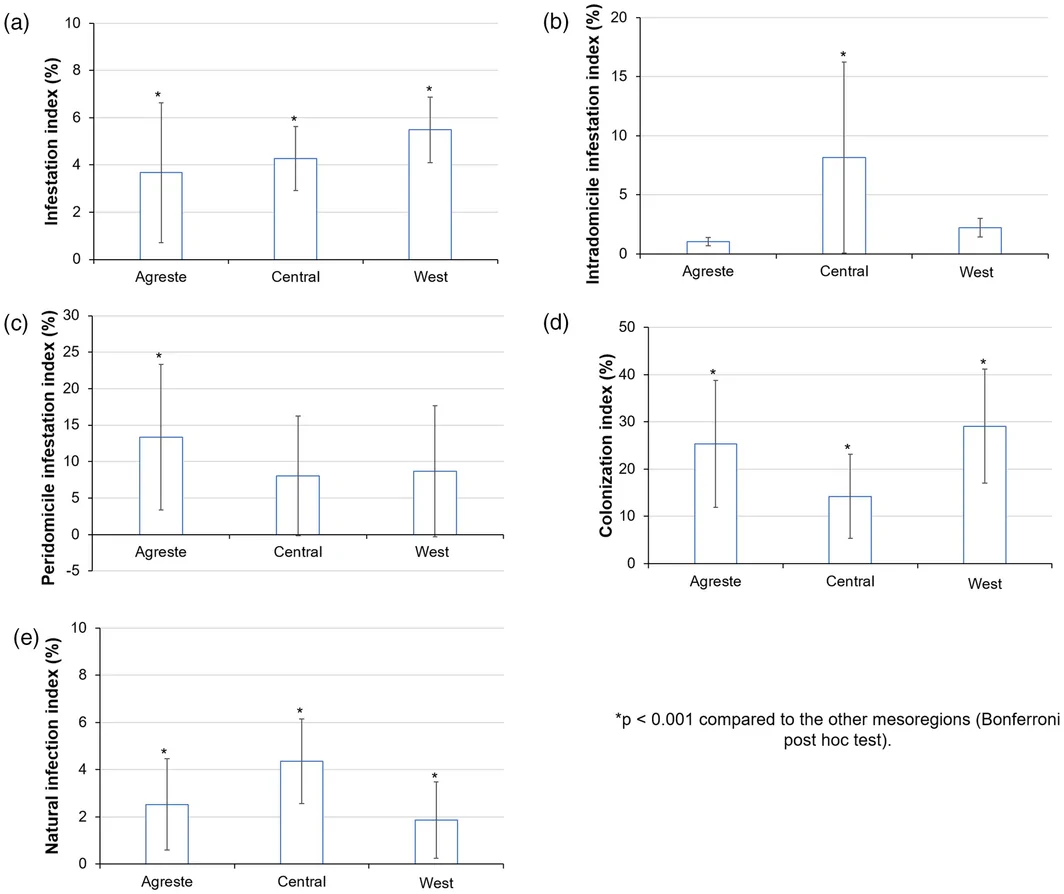
Entomological indicators for triatomines by mesoregion of the State of Rio Grande do Norte, Brazil, from 2005 to 2022.

The linear mixed‐effects regression model shown in Figure S1 identified that all five entomological indicators varied significantly between 2005 and 2022 (*p* < 0.001). Notably, the peridomiciliary infestation index was the only one to show a decreasing trend over the period (β = −0.616; 95% CI: −0.941 to −0.292; *p* < 0.001). In contrast, the other indicators, overall infestation (β = −0.069; 95% CI: −0.101 to −0.036; *p* < 0.001), intradomiciliary infestation (β = 0.305; 95% CI: 0.145–0.465; *p* < 0.001), colonization (β = 1.578; 95% CI: 1.398–1.758; *p* < 0.001) and natural infection by *T. cruzi* (β = 0.113; 95% CI: 0.083–0.144; *p* < 0.001) showed increasing trends over the study period.

The most frequently captured species across the three mesoregions was *T. brasiliensis* (*n* = 20,848), followed by *T. pseudomaculata* (*n* = 18,341), *R. nasutus* (*n* = 4465), *P. lutzi* (*n* = 3561) and *Triatoma petrocchiae* Pinto & Barreto (*n* = 182). The West mesoregion recorded the highest number of specimens captured (*n* = 24,874) and the greatest triatomine diversity, with five species documented. *Triatoma brasiliensis*, *T. pseudomaculata* and *R. nasutus* adults and nymphs were found in both intradomiciliary and peridomiciliary environments in the Agreste, Central, and West mesoregions. In addition, *P. lutzi* adults and nymphs were also found in the peridomicile of all three mesoregions (Table S3).

In terms of species distribution, *T. brasiliensis* and *T. pseudomaculata* were present in most municipalities where entomological surveillance was conducted in the Agreste, Central, and West mesoregions. Currais Novos, in the Central mesoregion, recorded the highest number of *T. brasiliensis*, *T. pseudomaculata*, *R. nasutus* and *P. lutzi* specimens captured, with triatomine counts ranging from 600 to 2000 insects. *Rhodnius nasutus* and *P. lutzi* were found in 79 and 92 municipalities that performed entomological surveillance, respectively. *Triatoma petrocchiae* was identified in 11 municipalities in the West mesoregion (Figure 1b–f).

## DISCUSSION

The entomological surveillance activities evaluated in this study revealed the exclusive presence of native triatomine species in the surveyed DUs, high colonisation indices, and a wide distribution of these vectors across municipalities in the West, Central, and Agreste mesoregions of RN state, highlighting the challenges in vector control in the semi‐arid region.

According to the findings, entomological surveillance activities were continuously conducted over the 13‐year period in only 15/142 municipalities, most of them located in the West mesoregion. Additionally, a significant proportion (46.5%) of municipalities in the Agreste mesoregion did not implement entomological surveillance activities during the study period. This situation is concerning because the absence of surveillance allows various triatomine species to infest and colonize internal and external environments in the DUs. This increases the likelihood of contact among vectors, humans, and domestic animals, consequently increasing the risk of *T. cruzi* transmission.

Despite the discontinuity of entomological surveillance activities in the state of RN, our findings suggest that chemical control measures were likely implemented effectively in most positive DUs across the three mesoregions. Several studies conducted in municipalities within the studied mesoregions have reported the presence of *T. cruzi* vectors in DUs (Barbosa‐Silva et al., 2016, 2019; Honorato et al., 2021; Lilioso et al., 2017; Sampaio et al., 2023), as well as in other states in Brazil's Northeast region (Ferreira et al., 2020; Lilioso et al., 2017; Oliveira et al., 2016). The high number of positive DUs indicates reinfestation once the residual effect of the insecticide wears off (Ceballos et al., 2011; Dumonteil et al., 2004). Furthermore, the persistent triatomine infestation in intradomicile and peridomicile environments, as well as high colonization indices in the Agreste, Central, and West mesoregions, indicate that the risk of vectorial transmission remains in these areas. Previous studies have also demonstrated a high infestation index in peridomestic areas of municipalities in the West mesoregion, particularly in ecotopes such as tile piles and chicken coops (Barbosa‐Silva et al., 2016; Honorato et al., 2021).

The West mesoregion showed the greatest diversity of triatomine species, as well as the highest infestation and colonization indices. However, it exhibited a lower natural infection index. In contrast, the Central mesoregion presented lower triatomine diversity but a higher *T. cruzi* natural infection index throughout most of the study period. This discrepancy may be explained by the greater number of entomological surveillance activities in the West mesoregion, which comprises more municipalities classified as medium‐ and high‐risk for *T. cruzi* transmission.

*Triatoma brasiliensis* and *T. pseudomaculata* were the species most frequently captured in intra‐ and peridomiciliary environments in the Agreste, Central, and West mesoregions throughout the study period. Between 1993 and 1999, Costa et al (Costa et al., 2003). reported a high density of *T. brasiliensis* across several states in northeastern Brazil, including RN state. Additionally, the authors also demonstrated a high colonization index of *T. brasiliensis* in anthropic environments, along with variable *T. cruzi* infection rates. Subsequently, several studies have also associated this species and *T. pseudomaculata* with the same environments in the state of RN (Araújo‐Neto et al., 2019; Barbosa‐Silva et al., 2016, 2019; Honorato et al., 2021; Lilioso et al., 2017; Lilioso, Reigada, et al., 2020; Lima‐Neiva et al., 2021; Sampaio et al., 2023) and in other states in the Northeast region, such as Ceará (Candido et al., 2019; Coutinho et al., 2014), Pernambuco (Silva et al., 2012, 2015), Paraíba (Lilioso et al., 2017; Oliveira et al., 2016) and Piauí (Ferreira et al., 2020) emphasizing the epidemiological relevance of these vectors in the *T. cruzi* transmission cycle and their adaptation to anthropic environments.

*Triatoma brasiliensis* and *T. pseudomaculata* are often found infesting and colonizing intra‐ and peridomiciliary environments. They frequently have high natural infection rates by *T. cruzi* (Costa et al., 2003; Sarquis et al., 2004). Studies conducted in the state of RN (Barbosa‐Silva et al., 2016, 2019; Honorato et al., 2021), Ceará (Parente et al., 2017; Sarquis et al., 2004, 2006, 2012) and Pernambuco (Silva et al., 2012, 2015, 2021, 2024) reported the predominance of these species in the peridomicile, corroborating our findings. *Triatoma brasiliensis* has demonstrated the ability to colonize a variety of ecotopes in different environments. It has been found in rocky outcrops, sylvatic environments, several peridomestic ecotopes, and inside dwellings (Barbosa‐Silva et al., 2016, 2019; Bezerra et al., 2014; Ribeiro et al., 2019). In contrast, *T. pseudomaculata* was primarily found in peridomicile areas, associated with birds (Barbosa‐Silva et al., 2016; Ferreira et al., 2020), and in the wild environment, inhabiting dry tree bark of various species (Carbajal‐de‐la‐Fuente et al., 2008; Freitas et al., 2005) and bird nests (Barbosa‐Silva et al., 2016; Carbajal‐de‐la‐Fuente et al., 2008). The presence of these species in DUs is concerning because several studies have reported a high natural infection index of *T. cruzi* in these vectors in various municipalities of the state of RN (Barbosa‐Silva et al., 2016; Honorato et al., 2021; Lilioso, Reigada, et al., 2020; Lima‐Oliveira et al., 2020).

*Rhodnius nasutus* and *P. lutzi* are widely distributed in the state, although they were captured less frequently. Their proximity to the wild environment likely attracts them to DUs by artificial light or in search of food, explaining their occasional occurrence in these units. Additionally, the use of carnauba palm (*Copernicia prunifera* Mill.) H.E.Moore (Arecales: Arecaceae) trunks to build peridomestic structures and handmade items contributes to the passive transport of *R. nasutus* from its natural habitat to DUs (Sarquis et al., 2004, 2006). *Rhodnius nasutus* was found in a dry carnauba palm thatch in the peridomicile environment of Apodi, which is located in the West mesoregion of RN (Barbosa‐Silva et al., 2016). In contrast, *P. lutzi* is predominantly observed in peridomicile environments across the three mesoregions and raises concern due to reports of high natural infection index by *T. cruzi* in intradomicile environments in RN.

*Triatoma petrocchiae* had limited distribution in the West mesoregion and was captured in low numbers. This species inhabits rocky outcrops in the northeastern semi‐arid region (Costa et al., 2014; Dale et al., 2018) and is rarely found in intra‐ or peridomestic areas (Lima‐Oliveira et al., 2020). Nevertheless, its presence has been documented in anthropic environments in RN, according to recent reports (Barbosa‐Silva et al., 2019; Carbajal‐de‐la‐Fuente et al., 2008), and in Paraíba (Lilioso, Pires‐Silva, et al., 2020), where it was found cohabiting rocky outcrops with *T. brasiliensis*. Therefore, continuous implementation of entomological surveillance is essential. The presence of these species demonstrates the diversity of triatomine fauna in the state of RN, as well as the dispersion of species across the Agreste, Central, and West mesoregions. This phenomenon can contribute to the *T. cruzi* transmission cycle.

In addition to the species *T. brasiliensis*, *T. pseudomaculata*, *P. lutzi*, *R. nasutus*, and *T. petrocchiae* identified in the present study, previous investigations indicate that the triatomine fauna of RN also includes *Panstrongylus diasi* Pinto & Lent, *P. megistus*, *Triatoma melanocephala* Neiva & Pinto, and *Triatoma rubrofasciata* De Geer (Barbosa‐Silva et al., 2019; Costa et al., 2003). Additionally, *Psammolestes tertius* Lent & Jurberg was recorded for the first time in a wild environment in the region (Barbosa‐Silva et al., 2018). More recently, *Triatoma chiarii*, a newly described species for the state, was documented in an anthropic environment (Barbosa‐Silva et al., 2026). Together, these findings expand current understanding of vector diversity and distribution in RN, underscoring the ecological complexity and epidemiological relevance of the local triatomine assemblage.

The results underscore the imperative to strengthen and maintain continuity in entomological surveillance activities, particularly in settings where the presence of triatomine vectors poses a considerable epidemiological threat. Furthermore, they highlight the critical role of robust, systematically structured, and sustained health education strategies that promote community‐level knowledge and health literacy, encourage active engagement of local populations, and support the adoption of sustainable preventive behaviours. Such educational interventions are essential not only for enhancing risk perception but also for enabling early vector identification by community members and reinforcing behavioural changes that effectively reduce transmission risk.

Multidisciplinary research focused on triatomine taxonomy, when integrated with advanced methodologies for *T. cruzi* detection, can substantially enhance the performance and responsiveness of surveillance systems. The coordinated integration of scientific research, operational surveillance, and comprehensive health education programs represents a strategic framework that positions communities as active co‐producers of health. This approach strengthens the long‐term, sustainable prevention and control of Chagas disease and contributes to more resilient public health infrastructures.

## CONCLUSION

The absence of continuous entomological surveillance activities has contributed to triatomine infestation and high colonization index in DUs located in the Agreste, Central, and West mesoregions of the state of RN. Additionally, the consistent presence of native species, such as *T. brasiliensis* and *T. pseudomaculata*, infected with *T. cruzi* in DUs, suggests a potential risk of parasite transmission to humans and domestic animals. These findings highlight the important role of native species in the semi‐arid zone in maintaining *T. cruzi* infections within anthropogenic environments.

## AUTHOR CONTRIBUTIONS

**Letícia Mikardya Lima Sales:** Conceptualization; data curation; formal analysis; investigation; methodology; writing – original draft; writing – review and editing. **Christiane Carlos Araújo de Negreiros:** Conceptualization; data curation; investigation; methodology; writing – original draft; visualization; writing – review and editing. **Lucas Abrantes Batista:** Formal analysis; writing – original draft; visualization; writing – review and editing. **George Harisson Felinto Sampaio:** Conceptualization; writing – original draft; visualization; writing – review and editing. **Nathan Ravi Medeiros Honorato:** Conceptualization; methodology; visualization; writing – review and editing. **Carlos Ramon do Nascimento Brito:** Conceptualization; methodology; visualization; writing – original draft; writing – review and editing; funding acquisition; supervision. **Rand Randall Martins:** Formal analysis; visualization; writing – review and editing; methodology. **Lúcia Maria Abrantes Aguiar:** Data curation; visualization; writing – review and editing; methodology. **Lúcia Maria da Cunha Galvão:** Supervision; writing – review and editing; conceptualization; visualization. **Antonia Claudia Jácome da Câmara:** Conceptualization; investigation; writing – review and editing; visualization; project administration; supervision. **Andressa Noronha Barbosa‐Silva Carvalho:** Conceptualization; data curation; investigation; methodology; visualization; project administration; writing – review and editing.

## FUNDING INFORMATION

This work was supported by research grants from the National Council for Scientific and Technological Development (CNPq) *MCTI/FNDCT* number 22/2024 (CRNB). LMLS, LAB, NRMH and CCAN received Doctoral and Master's scholarships from the *Coordenação de Aperfeiçoamento de Pessoal de Nível Superior‐CAPES*, ANBS and GHFS received Postdoctoral scholarships from the *Conselho Nacional de Desenvolvimento Científico e Tecnológico MCTI*/*CNPq*, and LMCG is a *CNPq* fellow. The Article Processing Charge (APC) for the publication of this research was covered by the Coordination for the Improvement of Higher Education Personnel‐CAPES (ROR: 00 × 0ma614), and a Creative Commons Attribution (CC BY) licence was applied to the Accepted Manuscript.

## CONFLICT OF INTEREST STATEMENT

The authors declare no conflicts of interest.

## Supporting information


**Appendix S1.** Structured reflexivity statement for international research partnerships.

**Figure S1.** Linear mixed‐effects regression in entomological indicators for triatomines in Rio Grande do Norte, Brazil, between 2005 and 2022.

**Table S1.** Entomological surveillance activities and presence of triatomines in domiciliary units in municipalities in the mesoregions of Rio Grande do Norte from 2005 to 2022.
**Table S2.** Number of domiciliary units searched, positive for triatomines and sprayed by mesoregion in Rio Grande do Norte, from 2005 to 2022.
**Table S3.** Triatomine species captured by the environment in the different mesoregions of the state of Rio Grande do Norte, from 2005 to 2022.

## Data Availability

All data are openly available in Dryad Repository. The dataset is available as follows: https://doi.org/10.5061/dryad.c866t1gnt (Sales et al., 2026).
