# Supplementary material for: Entomological surveillance of Trypanosoma cruzi vectors in Rio Grande do Norte, Brazil
Source: Med Vet Entomol. 2026 Jun 26;40(4):1121–9. doi: 10.1111/mve.70093 (PMC13749608; doi:10.1111/mve.70093)
Supplement: Supplementary file 1 [file MVE-40-1121-s001.docx]

|  | **Number of municipalities (%)** | | | | | | | | | | | |
| --- | --- | --- | --- | --- | --- | --- | --- | --- | --- | --- | --- | --- |
| **Years of execution** | **Agreste mesoregion** | | | | **Central mesoregion** | | | | **West mesoregion** | | | |
|  | **ES (%)** | | **PT (%)** | | **ES (%)** | | **PT (%)** | | **ES (%)** | | **PT (%)** | |
| 12 | 2 | (4.7) | 2 | (8.7) | 4 | (10.9) | 4 | (13.3) | 9 | (14.5) | 9 | (17.0) |
| 10 | 8 | (18.6) | 8 | (34.8) | 3 | (8.1) | 3 | (10.0) | 11 | (17.7) | 11 | (20.7) |
| 8 | 2 | (4.7) | 2 | (8.7) | 6 | (16.2) | 6 | (20.0) | 15 | (24.2) | 15 | (28.3) |
| 6 | 4 | (9.3) | 4 | (17.4) | 10 | (27.0) | 10 | (33.3) | 5 | (8.1) | 5 | (9.4) |
| 4 | 5 | (11.6) | 5 | (21.7) | 5 | (13.5) | 5 | (16.7) | 10 | (16.1) | 10 | (18.9) |
| 2 | 2 | (4.7) | 2 | (8.7) | 2 | (5.4) | 2 | (6.7) | 10 | (16.1) | 10 | (18.9) |
| None | 20 | (46.5) | - | | 7 | (18.9) | - | | 7 | (11.3) | - | |
| Total | 43 | (100.0) | 23 | (53.5) | 37 | (100.0) | 30 | (81.1) | 62 | (100.0) | 60 | (96.8) |

**Table S1.** Entomological surveillance activities and presence of triatomines in domiciliary units in municipalities in the mesoregions of Rio Grande do Norte, Brazil, from 2005 to 2022

ES: Entomological surveillance; PT: Presence of triatomines.

**Table S2.** Number of domiciliary units searched, positive for triatomines and sprayed by mesoregion in Rio Grande do Norte, Brazil, from 2005 to 2022

| **Year** | **Agreste Mesoregion** | | | | **Central Mesoregion** | | | | **West Mesoregion** | | | |
| --- | --- | --- | --- | --- | --- | --- | --- | --- | --- | --- | --- | --- |
|  | **Searched** | **Positives** | **Sprayed** | **(%)** | **Searched** | **Positives** | **Sprayed** | **(%)** | **Searched** | **Positives** | **Sprayed** | **(%)** |
| **2005** | 12,029 | 354 | 346 | (97.7) | 17,237 | 1,092 | 1,432 | (131.1) | 38,497 | 1,842 | 2,031 | (110.3) |
| **2006** | 14,002 | 1,766 | 434 | (24.6) | 26,014 | 1,088 | 1,417 | (130.3) | 28,997 | 1,791 | 1,655 | (92.4) |
| **2007** | 10,066 | 337 | 308 | (91.4) | 35,950 | 1,261 | 1,822 | (144.5) | 30,225 | 1,235 | 1,200 | (97.2) |
| **2008** | 5,446 | 52 | 52 | (100.0) | 17,148 | 734 | 981 | (133.6) | 22,660 | 1,504 | 1,224 | (81.4) |
| **2009** | 9,242 | 188 | 217 | (115.4) | 17,772 | 592 | 675 | (114.0) | 24,360 | 1,047 | 997 | (95.2) |
| **2010** | 10,994 | 375 | 337 | (89.9) | 19,775 | 1,208 | 1,099 | (90.8) | 22,273 | 1,109 | 1,002 | (90.3) |
| **2011** | 9,177 | 266 | 220 | (82.7) | 9,434 | 470 | 262 | (55.7) | 21,318 | 1,156 | 1,064 | (92.0) |
| **2012** | 6,182 | 127 | 110 | (86.6) | 22,360 | 1,009 | 917 | (90.9) | 16,195 | 876 | 801 | (91.4) |
| **2013** | 5,132 | 188 | 181 | (96.3) | 18,882 | 657 | 509 | (77.5) | 20,975 | 1,229 | 940 | (76.5) |
| **2014** | 6,684 | 199 | 169 | (84.9) | 15,325 | 567 | 491 | (86.6) | 20,924 | 1,181 | 984 | (83.3) |
| **2015** | 3,966 | 131 | 151 | (115.3) | 22,828 | 1,240 | 1,114 | (89.8) | 16,451 | 1,513 | 1,104 | (73.0) |
| **2019** | 1,360 | 17 | 20 | (117.6) | 15,426 | 338 | 315 | (93.2) | 5,630 | 210 | 144 | (68.6) |
| **2022** | NR | NR | NR | NR | 14,108 | 221 | 179 | (81.0) | NR | NR | NR | NR |
| **Total** | 94,280 | 4,000 | 2,545 | (63.6) | 252,259 | 10,477 | 11,213 | (107.0) | 268,505 | 11,693 | 13,146 | 112.4 |

NR: Not Realized

**Table S3**. Triatomine species captured by environment in the different mesoregions of the state of Rio Grande do Norte, Brazil, from 2005 to 2022

| **Species** | **Intradomicile** | | | | **Peridomicile** | | | | **Total** | |
| --- | --- | --- | --- | --- | --- | --- | --- | --- | --- | --- |
|  | **A** | **N** | **T** | **%** | **A** | **N** | **T** | **%** | **No.** | **%** |
| **Agreste Mesoregion** | | | | | | | | | | |
| *Triatoma brasiliensis* | 277 | 125 | 402 | 51.7 | 1,072 | 520 | 1,592 | 35.3 | 1,994 | 37.7 |
| *Triatoma pseudomaculata* | 179 | 124 | 303 | 38.9 | 1,148 | 453 | 1,601 | 35.5 | 1,904 | 36.0 |
| *Panstrongylus lutzi* | 0 | 2 | 2 | 0.3 | 512 | 126 | 638 | 14.1 | 640 | 12.1 |
| *Rhodnius nasutus* | 33 | 38 | 71 | 9.1 | 453 | 225 | 678 | 15.0 | 749 | 14.2 |
| **Total** | **489** | **289** | **778** | **100.0** | **3,185** | **1,324** | **4,509** | **100.0** | **5,287** | **100.0** |
| **Central Mesoregion** | | | | | | | | | | |
|  | | | | | | | | | | |
| *Triatoma brasiliensis* | 1,042 | 427 | 1,469 | 52.3 | 3,943 | 1,607 | 5,550 | 38.5 | 7,019 | 40.7 |
| *Triatoma pseudomaculata* | 912 | 307 | 1,219 | 43.4 | 3,698 | 1,372 | 5,070 | 35.1 | 6,289 | 36.5 |
| *Panstrongylus lutzi* | 0 | 3 | 3 | 0.1 | 1,342 | 415 | 1,757 | 12.2 | 1,760 | 10.2 |
| *Rhodnius nasutus* | 50 | 70 | 120 | 4.3 | 1,487 | 561 | 2,048 | 14.2 | 2,168 | 12.6 |
| **Total** | **2,004** | **807** | **2,811** | **100.0** | **10,470** | **3,955** | **14,425** | **100.0** | **17,236** | **100.0** |
| **West Mesoregion** | | | | | | | | | | |
| *Triatoma brasiliensis* | 2,029 | 571 | 2,600 | 54.9 | 6,488 | 2,747 | 9,235 | 45.9 | 11,835 | 47.6 |
| *Triatoma pseudomaculata* | 1,455 | 507 | 1,962 | 41.5 | 5,946 | 2,240 | 8,186 | 40.6 | 10,148 | 40.8 |
| *Panstrongylus lutzi* | 0 | 32 | 32 | 0.7 | 913 | 216 | 1,129 | 5.6 | 1,161 | 4.7 |
| *Rhodnius nasutus* | 25 | 102 | 127 | 2.7 | 1,157 | 264 | 1,421 | 7.1 | 1,548 | 6.2 |
| *Triatoma petrocchiae* | 10 | 2 | 12 | 0.3 | 107 | 63 | 170 | 0.8 | 182 | 0.7 |
| **Total** | **3,519** | **1,214** | **4,733** | **100.0** | **14,611** | **5,530** | **20,141** | **100.0** | **24,874** | **100.0** |

A: adult; N: nymphs; T: total; %: percentage; No.: number of specimens captured.
