# Supplementary material for: Entomological surveillance of Trypanosoma cruzi vectors in Rio Grande do Norte, Brazil
Source: Med Vet Entomol. 2026 Jun 26;40(4):1121–9. doi: 10.1111/mve.70093 (PMC13749608; doi:10.1111/mve.70093)
Supplement: Supplementary file 2 [file MVE-40-1121-s002.docx]

**Figure S1.** Linear mixed-effects regression in entomological indicators for triatomines in Rio Grande do Norte, Brazil, between 2005 and 2022.
