# Supplementary material for: Entomological surveillance of Trypanosoma cruzi vectors in Rio Grande do Norte, Brazil
Source: Med Vet Entomol. 2026 Jun 26;40(4):1121–9. doi: 10.1111/mve.70093 (PMC13749608; doi:10.1111/mve.70093)
Supplement: Supplementary file 3 [file MVE-40-1121-s003.docx]

STRUCTURED REFLEXIVITY STATEMENT FOR INTERNATIONAL RESEARCH PARTNERSHIPS

Please complete all sections relevant to your research study

| Study conceptualisation |  |
| --- | --- |
| Please justify the choice to conduct the study in  and/or acquire samples from the LMIC location | This study was conceived, conducted, and funded entirely within Brazil by Brazilian institutions. The choice of location reflects the epidemiological relevance of the research topic in the Brazilian context, as well as the availability of appropriate infrastructure, expertise, and study populations. All samples were collected locally in accordance with national regulations and ethical standards. |
| How does this study address local research priorities and how were local researchers  involved in study design? | This study directly addresses local research priorities by focusing on health issues that are highly relevant to the regional context. The research team is entirely composed of local professionals, including academic researchers, a public health surveillance manager, and graduate students, ensuring strong alignment with local needs and priorities. Local researchers were actively involved in all stages of the study, including conceptualization, study design, implementation, data analysis, and interpretation of the results. This comprehensive involvement ensured that the study was contextually appropriate and responsive to local public health demands. |
| Research management |  |
| How has funding been used to support the local  research team(s)? | This work was supported by research grants from the Conselho Nacional de Desenvolvimento Científico e Tecnológico (MCTI/FNDCT; grant number 22/2024, CRNB), which were used to support field activities, including per diem and travel expenses associated with data collection. In addition, members of the local research team received academic funding through national agencies: LMLS, LAB, NRMH, and CCAN were supported by Master’s and Doctoral scholarships from the Coordenação de Aperfeiçoamento de Pessoal de Nível Superior (CAPES); ANBS and GHFS received postdoctoral fellowships from CNPq; and LMCG is a CNPq research fellow.  This funding structure contributed directly to strengthening local research capacity by supporting personnel training and the overall execution of the study within the local context. |
| Data acquisition and analysis |  |
| How are research staff who conducted data collection acknowledged? | All research staff involved in data collection are appropriately acknowledged through authorship and/or in the acknowledgments section, in accordance with their level of contribution. The team includes professors, researchers, and graduate students (Master’s and Doctoral scholarship holders), whose roles in data collection and study implementation are duly recognized. |
| How have members of the research partnership been provided with access to study data and  analytical tools? | Study data were made available by the State Secretariat of Public Health of Rio Grande do Norte, in collaboration with municipal health authorities and local health agents. All members of the research partnership were granted appropriate access to the dataset and analytical tools required for the study, in accordance with their roles and responsibilities, ensuring collaborative data analysis and interpretation. |
| If genetic resources were shared, how were the principles of the Nagoya Protocol on the  equitable sharing of benefits | Not applicable, as no genetic resources were accessed, collected, or shared in this study. |
| Data interpretation |  |
| How have research partners collaborated in  interpreting study data? | Research partners collaborated in the interpretation of study data through a series of meetings and discussions involving professors, researchers, and public health practitioners (including endemic disease control managers). These interactions enabled the integration of scientific expertise with practical field knowledge, contributing to a contextually informed and robust interpretation of the findings. |
| Drafting and revising for intellectual content |  |
| How were research partners supported to develop writing skills? | Research partners were supported in developing scientific writing skills through active participation in all stages of manuscript preparation. The first author, Letícia M. L. Sales, a Master’s scholarship holder, led data organization and initial drafting of the manuscript, while senior researchers provided guidance on data interpretation, contextualization, and scientific writing. This collaborative process was embedded within an established research group with over 20 years of experience in the field, which is also actively engaged in training and mentoring human resources, thereby strengthening local capacity in scientific communication. |
| Authorship |  |
| How is the leadership, contribution and ownership of this work by LMIC researchers recognised within the authorship? | The leadership, contribution, and ownership of this work by LMIC researchers are reflected in the authorship structure. The study was conceived, led, and conducted by a local research group with over 20 years of experience in the field. LMIC researchers hold key authorship positions, including first and senior authorship, and were responsible for study design, data collection, analysis, and manuscript preparation. This group is also actively engaged in training and mentoring human resources, further reinforcing local ownership and capacity building. |
| How have early career researchers across the partnership been included within the authorship team? | Early career researchers were actively included in the authorship team through meaningful contributions across all stages of the study. LMLS, LAB, NRMH, and CCAN (Master’s and Doctoral scholarship holders), as well as ANBS and GHFS (postdoctoral researchers), were directly involved in data collection, analysis, and manuscript preparation, and are therefore included as authors. Their participation provided opportunities for leadership development and professional training. Senior researchers, including LMCG (with over 40 years of experience), ACJC (over 20 years), and CRNB (over 10 years), provided supervision, mentorship, and guidance, ensuring both scientific rigor and capacity building within the team. |
| How has gender balance been addressed within the authorship? | The authorship team demonstrates a balanced gender representation. The study is led by both female (LMCG, ACJC) and male (CRNB, RRM) senior researchers, reflecting balanced leadership. In addition, the team includes postgraduate researchers with a relatively balanced gender distribution (two women and three men), as well as additional researchers, contributing to an inclusive and diverse authorship. This composition reflects an effort to promote gender equity across different career stages within the research team. |
| Training |  |
| How has the project contributed to training of LMIC researchers? | This study was conducted and funded entirely within Brazil by Brazilian institutions and has directly contributed to the training of LMIC researchers. The project involved Master’s, Doctoral, and postdoctoral researchers who actively participated in all stages of the study, including fieldwork, data analysis, and manuscript preparation. In addition to generating scientific knowledge on the epidemiology and distribution of triatomines in the state of Rio Grande do Norte, the project aimed to strengthen local capacity by training qualified human resources and supporting evidence-based decision-making for vector control by state and municipal health authorities. |

Adapted from: Morton et al. 2021 “Consensus statement on measures to promote equitable authorship in the publication of research from international partnerships” <https://doi.org/10.1111/anae.15597>
